# Association between postoperative nutritional status and time to initiation of adjuvant chemotherapy in gastrointestinal cancers: a restricted cubic spline analysis

**DOI:** 10.3389/fnut.2026.1896619

**Published:** 2026-08-31

**Authors:** Na Liu, Sheng Sun, Xin Guan, Qian Xu, Yu Zhang, XiaoXiang Liu, Bolun Zhao

**Affiliations:** 1 School of Nursing, Dalian Medical University, Dalian, Liaoning, China; 2 The Second Affiliated Hospital of Dalian Medical University, Dalian, Liaoning, China

**Keywords:** gastrointestinal cancer, influencing factors, nutritional status, restricted cubic spline, time to chemotherapy

## Abstract

**Purpose:**

To explore the relationship between early postoperative nutritional status and time-to-chemotherapy (TTC) in gastrointestinal cancer patients and provide a reference for nutritional intervention and reasonable chemotherapy arrangements.

**Methods:**

This study enrolled consecutive patients who underwent radical gastrointestinal cancer surgery with planned adjuvant chemotherapy at a tertiary hospital in Dalian, China (June 2023–June 2025). Nutritional indicators included BMI, NRS-2002 score, and serum albumin concentration and their associations with TTC were analyzed using restricted cubic splines (RCS) and multivariable regression models.

**Results:**

RCS analysis revealed an approximately linear dose–response relationship between the NRS-2002 score and TTC. Based on multivariate regression, each 1-point increase prolonged TTC by 2.36 days and conferred a 57% higher risk of chemotherapy delay. Conversely, BMI and serum ALB levels exhibited nonlinear associations with TTC (U-shaped and inverted L-shaped, respectively). Exploratory inflection points derived from RCS were BMI ≥ 23.82 kg/m^2^ and serum albumin ≥38.37 g/L. Multivariable-adjusted associations suggested that patients above these inflection points tended to have a 4-day shorter TTC, with a 42% reduction in chemotherapy-delay risk (BMI only).

**Conclusion:**

Postoperative nutritional indicators are closely related to TTC. The inflection point values of BMI and serum albumin derived from this study are hypothesis-generating findings and should not be regarded as validated clinical targets for routine clinical application. Targeted nutritional management may help optimize chemotherapy timing and guide clinical nursing work.

## 1 Introduction

Gastrointestinal cancers are currently the single most common cancer worldwide (1). Radical surgical resection remains the cornerstone and most effective treatment for gastrointestinal cancers. Surgical stress may influence metastatic processes in many ways, and surgery alone does not prevent cancer recurrence (2). Postoperative adjuvant chemotherapy can eradicate residual micrometastases and reduce the probability of recurrence. It is well known internationally as a powerful component of therapy, and as a combination treatment for gastrointestinal cancers, its benefits have been established (3). Existing studies have shown that adjuvant chemotherapy following radical resection improves overall survival, effectively reduces recurrence rates, and plays a major role in improving patient prognosis (4). However, waiting too long before the first postoperative chemotherapy increases the risk of micrometastatic progression, and a long time-to-chemotherapy (TTC) after surgery has adverse effects on overall survival (5, 6). Therefore, the timing of the first postoperative chemotherapy is important.

TTC refers to the interval from a patient’s surgical treatment to the initiation of the first adjuvant chemotherapy (7). International recommendations for the timing of the first postoperative first chemotherapy in gastrointestinal cancer are inconsistent: the European Society for Medical Oncology (ESMO) guideline suggests starting adjuvant chemotherapy within 8 weeks after surgery (8), whereas Chinese guidelines recommend initiating adjuvant chemotherapy at approximately 4 weeks post-operatively (9). A shortened interval to first chemotherapy may be detrimental to the recovery of gastrointestinal function and overall physical condition, potentially leading to poorer tolerance of adjuvant chemotherapy. Unlike patients with other malignancies, those who have undergone abdominal surgery typically require a dietary transition period and therefore need a longer convalescence; this does not imply, however, that adjuvant chemotherapy can be commenced at any time without consequence. Adjuvant chemotherapy for gastrointestinal cancer to be started within 6–8 weeks after surgery is recommended (7). Some studies have indicated that the interval from surgery to adjuvant chemotherapy is critical for key oncologic outcomes, with earlier chemotherapy significantly prolonging progression-free survival, reducing recurrence rates, and improving health-related quality of life (4, 10). Therefore, promoting the early initiation of adjuvant chemotherapy can improve patient prognosis and quality of survival. Current research specifically addressing the timing of first postoperative chemotherapy in gastrointestinal cancer is limited; most studies have focused on chemotherapy regimens, efficacy, and adverse-event-related prognostic factors (11–14), whereas factors influencing time to first chemotherapy have been less frequently examined. Early enteral nutrition not only accelerates postoperative recovery of gastrointestinal function and shortens hospital stay but also helps secure time for subsequent adjuvant chemotherapy; conversely, prolonged postoperative hospitalization, prolonged operative time, and intraoperative blood loss may delay chemotherapy initiation by exacerbating systemic inflammation and nutritional depletion, thereby impairing recovery (15, 16). GANENKO et al. found that age, race, and hospital type were all significantly associated with whether adjuvant chemotherapy was administered in a timely manner (17). Nevertheless, there is no consensus regarding how perioperative events and individual patient characteristics independently influence the timing of chemotherapy. Global studies report that most cancer patients exhibit metabolic disturbances, with only 4% of patients showing metabolic parameters within normal ranges (18). As a disease associated with high metabolic demands, cancer is frequently accompanied by malnutrition, which is highly prevalent among oncology populations and worsens during the course of anticancer treatment. This condition not only increases health care costs but also reduces patients’ quality of life with increases mortality risk (19, 20). Patients with gastrointestinal cancer experience impaired digestion and absorption due to alterations in alimentary tract physiology. Moreover, surgery for malignant gastrointestinal tumors imposes significant physiological stress (21), and unstable postoperative nutritional status is common. The incidence of malnutrition ranges from 20 to 70%, which increases the risk of postoperative complications and thereby affects recovery of the patients (22–24). Nutritional status spans the preoperative-postoperative-chemotherapy continuum: Nutritional reserves before the first chemotherapy session determine the nutritional trajectory throughout the chemotherapy period (25), and during chemotherapy, patients’ tolerance, prognosis, and quality of life are closely associated with nutritional status (26). Nutrition is a key, modifiable factor during treatment, and therefore is amenable to clinical intervention (27). Whether early postoperative nutritional status influences the timing of the first chemotherapy and thereby exerts cascading effects on the efficacy of adjuvant chemotherapy remains unclear.

This study employed restricted cubic spline (RCS) analysis combined with multivariable linear and logistic regression models to systematically investigate the association and dose–response relationship between early postoperative nutritional status and TTC in patients with gastrointestinal cancer. The objective was to identify nutritional risk factors affecting TTC and to explore potential inflection point effects. These findings highlight the potential value of proactive nutritional management during the early postoperative period in clinical practice. By improving early postoperative nutritional reserves and shortening the recovery period, patients’ nutritional status can reach targets that allow the initiation of chemotherapy on schedule. Through the optimized management of TTC, we aim to further improve patient survival and quality of life.

## 2 Methods

### 2.1 Study population

**Figure 1:**
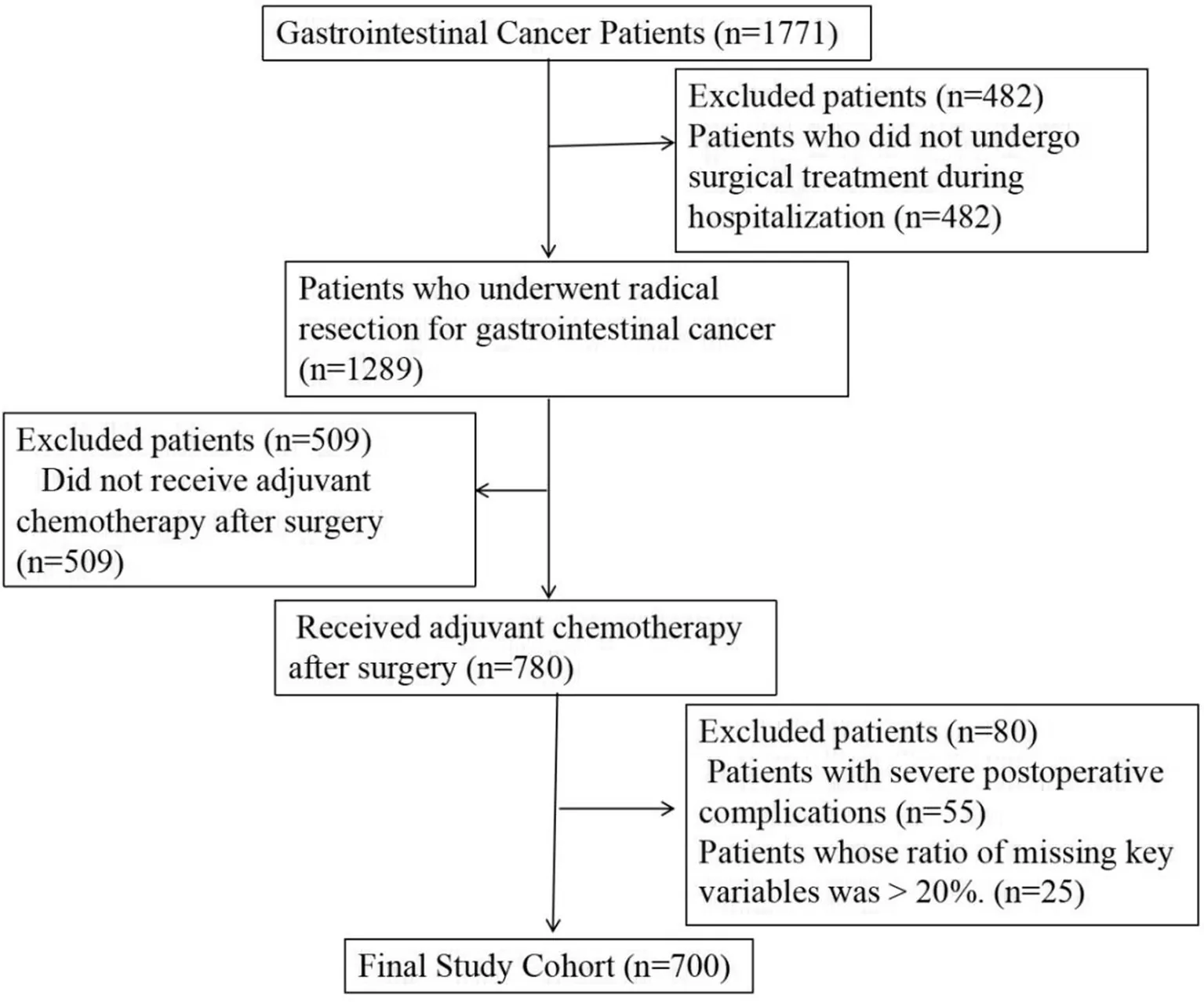
Flowchart of patient selection.

This retrospective study was approved by the Ethics Committee of a tertiary hospital (KY2025-746-01). The study population consisted of patients who underwent surgery in the Department of Gastrointestinal Surgery at a tertiary hospital in Dalian and who received adjuvant chemotherapy between June 2023 and June 2025 (Figure 1).

The inclusion criteria were as follows: ① patients who underwent radical resection for gastrointestinal cancer and received adjuvant chemotherapy after surgery (9, 28), ② patients without severe complications (29), and ③ patients with complete clinical data and informed consent.

The exclusion criteria were as follows: ① patients who did not undergo surgical treatment during hospitalization or who did not receive adjuvant chemotherapy after surgery, ② patients with severe postoperative complications, and ③ patients whose ratio of missing key variables was > 20%.

### 2.2 Data collection

Two researchers from the research team served as investigators to identify inpatient course records in the electronic medical record system (Epic Systems) and collect the required data. Postoperative BMI, serum hemoglobin concentration, serum albumin concentration and NRS 2002 score were uniformly collected between postoperative Days 3 and 7. This standardized time window was used for all participants to minimize measurement heterogeneity resulting from variable postoperative stress responses (30, 31). For the extracted independent demographic and clinical variables, a total of 17 items of data were collected in this study:

1. Patient indicators included age, sex, type of medical insurance, educational background, marital status, BMI (32), number of chronic diseases (33, 34), total length of hospital stay, length of postoperative hospital stay (35), time to resume diet after surgery, TTC, etc.

2. Surgical-related factors included surgical duration, surgical approach, blood loss, etc.

3. Laboratory indicators included serum albumin (ALB) and hemoglobin (HGB).

4. Scoring: Postoperative NRS-2002 nutritional score (36).

### 2.3 Statistical analysis

All collected data were subjected to dual independent entry and logical verification using Excel 2019 (Microsoft Corporation, United States) for initial processing. Patients whose key variables were missing more than 20% of the data were excluded. All statistical analyses were performed in R (version 4.0.5) within RStudio (version 1.4.1106) using complete-case analysis (37). Continuous variables are presented as the mean ± standard deviation (M ± SD) and were assessed for normality using the Shapiro–Wilk test and for homogeneity of variance using Levene’s test; categorical variables are expressed as counts (percentages) [n (%)]. Chi-square tests were used to compare chemotherapy delay rates (≥6 weeks vs. <6 weeks) across different nutritional groups. Pearson correlation was used to assess correlations between continuous variables. Chi-square tests were used for comparisons of categorical variables, whereas one-way ANOVA or t tests were used for continuous variables. A two-sided *p* < 0.05 was considered to indicate statistical significance. Dose–response relationships between BMI, serum albumin concentration, nutritional score and TTC were examined using restricted cubic spline plots generated with the rms and ggplot2 packages, adjusting for other significant covariates. The optimal knot positions were selected on the basis of model fitness principles, with 3 knots placed at the 10th, 50th, and 90th percentiles. Likelihood-ratio tests indicated significant nonlinear association for one variable and borderline-significant nonlinear association for the other (*p* = 0.05). Model diagnostics confirmed an acceptable residual distribution, stable model performance, and no influential outliers, indicating reliable and robust fitting results of the RCS model. Inflection points were numerically identified at positions where the slope of the smoothed RCS exposure-response curve changed markedly, yielding values of 23.82 kg/m^2^ for BMI and 38.37 g/L for serum albumin. These values represent exploratory inflection points derived from the current dataset and were used only for exploratory stratification within the present study. Multivariate linear regression models were constructed with BMI, serum albumin concentration, and nutritional score as independent variables and time to first chemotherapy as the continuous dependent variable. Logistic regression models were constructed with chemotherapy delay (≥6 weeks vs. <6 weeks) as the binary dependent variable. Finally, subgroup analyses were performed, and patients were stratified by gastric and intestinal diseases.

## 3 Results

In this study, 700 patients who underwent radical resection for gastrointestinal cancer followed by adjuvant chemotherapy were included. Among them, 264 patients (37.7%) had NRS-2002 scores ≥3, indicating a risk of malnutrition. After stratification by nutritional status, TTC was significantly longer in the high nutritional risk group, with a mean of 40.16 ± 18.86 days, than in the low nutritional risk group, which was 35.56 ± 13.30 days (Table 1). The rate of delayed postoperative chemotherapy (≥6 weeks) among patients with NRS-2002 scores ≥3 was 34.1%, which was significantly greater than that among patients with scores <3 (19.5%) (Table 2). Univariate analysis revealed that the NRS-2002 score, age, postoperative length of stay, total length of stay, time to postoperative dietary recovery, operative time, and intraoperative blood loss were significantly associated with delayed initiation of first chemotherapy (all *p* < 0.01). Serum albumin levels, HGB levels, and BMI were negatively correlated with TTC. Sex, education level, marital status, type of medical insurance, surgical procedure, and number of chronic diseases were not significantly associated with TTC (*p* > 0.05) (Table 3). RCS analysis indicated that BMI and serum albumin concentration were nonlinearly related to TTC and presented U-shaped and inverted L-shaped patterns, respectively, whereas the NRS-2002 nutritional score was linearly related to TTC.

**Table 1:** Baseline characteristics of patients in different nutritional groups (NRS2002) (*n* = 700).

Variable		<3,436 (62.3%)	≥3,264 (37.7%)
Sex n(%)	Male(0)	295(42.14%)	167 (23.86%)
	Female(1)	141(20.14%)	97 (13.86%)
Educational background n(%)	Primary school (0)	36 (5.14%)	37 (5.29%)
	Junior high school (1)	139(19.86%)	77 (11.00%)
	Senior high school (2)	88 (12.57%)	41 (5.86%)
	University or above (3)	173(24.71%)	109(15.57%)
Marital status n(%)	Married (0)	422(60.29%)	258(36.85%)
	Others (1)	14 (2.00%)	6 (0.86%)
Type of medical insurance n(%)	Basic medical insurance (0)	352(50.29%)	229(32.71%)
	Urban and rural resident basic medical insurance (1)	84 (12%)	35 (5.00%)
Surgical Approach n(%)	Intestinal (0)	271(38.72%)	165(23.57%)
	Gastric (1)	159(22.71%)	105(15.00%)
Age (M ± SD)		60.22 ± 7.96	69.97 ± 8.33
BMI (M ± SD)		24.10 ± 3.51	23.67 ± 3.38
Total length of hospital stay (M ± SD)		14.00 ± 4.11	14.53 ± 4.48
Length of postoperative hospital stay (M ± SD)		8.93 ± 2.97	9.38 ± 3.20
Time to resume diet after surgery (M ± SD)		3.72 ± 2.60	3.99 ± 2.93
Number of chronic diseases (M ± SD)		0.59 ± 0.77	0.90 ± 0.93
HGB (M ± SD)		127.29 ± 25.51	118.94 ± 26.89
ALB (M ± SD)		40.20 ± 4.51	38.91 ± 4.80
Surgical duration (M ± SD)		184.78 ± 60.07	185.06 ± 67.09
Blood loss (M ± SD)		52.63 ± 71.29	62.42 ± 87.08
TTC (M ± SD)		35.56 ± 13.30	40.16 ± 18.86

**Table 2:** Analysis results of time to first postoperative chemotherapy in different nutritional groups.

		<6 weeks〔n(%)〕	≥6 weeks〔n(%)〕	*P*
NRS2002	<3分	351 (80.5)	85 (19.5)	<0.01^**^
	≥3分	174 (65.9)	90 (34.1)	

**P < 0.01, statistically significant difference; *p < 0.05, statistically significant difference.

**Table 3:** Results of univariate analysis.

	Variable	TTC(d)	*T*	*P*
Sex	Male (462)	37.04 ± 14.41	−0.56	0.57
	Female (238)	37.75 ± 18.21		
Educational background	Primary school (73)	41.63 ± 21.70	−1.35	0.18
	Junior high school (216)	36.23 ± 12.30		
	Senior high school (129)	38.04 ± 14.82		
	University or above (282)	36.61 ± 16.67		
Marital status	Married (680)	37.42 ± 15.96	−1.35	0.18
	Others (20)	32.60 ± 7.12		
Type of medical insurance	Basic medical insurance (581)	37.04 ± 14.34	−0.90	0.37
	Urban and rural resident basic medical insurance (119)	38.46 ± 21.58		
Surgical approach	Intestinal (430)	36.38 ± 14.73	1.91	0.06
	Gastric (270)	38.72 ± 17.28		
NRS2002	<3(436)	35.54 ± 13.33	3.77	<0.01^**^
	≥3(264)	40.16 ± 18.86		
ALB	<38.37(245)	41.13 ± 21.57	−4.79	<0.01^**^
	≥38.37(455)	35.23 ± 11.00		
BMI	<23.82(328)	39.23 ± 19.92	−4.12	<0.01^**^
	≥23.82(372)	35.01 ± 10.39		
Age			4.33	<0.01^**^
Total length of hospital stay			4.32	<0.01^**^
Number of chronic diseases			−0.04	0.99
Length of postoperative hospital stay			5.37	<0.01^**^
Time to Resume diet after surgery			3.00	<0.01^**^
HGB			−2.10	0.04^*^
Surgical duration			3.95	<0.01^**^
blood loss			4.51	<0.01^**^

**P < 0.01, statistically significant difference; *p < 0.05, statistically significant difference.

### 3.1 Analysis of chemotherapy delay incidence across different nutritional groups

Patients with an NRS-2002 score ≥ 3 had a postoperative chemotherapy delay rate (≥ 6 weeks) of 34.1%, which was significantly greater than that of patients with a score < 3 (19.5%), and the difference was statistically significant (*p* < 0.01) (Table 2).

### 3.2 Associations between TTC and BMI, serum albumin, and NRS-2002 scores: RCS analysis

**Figure 2:**
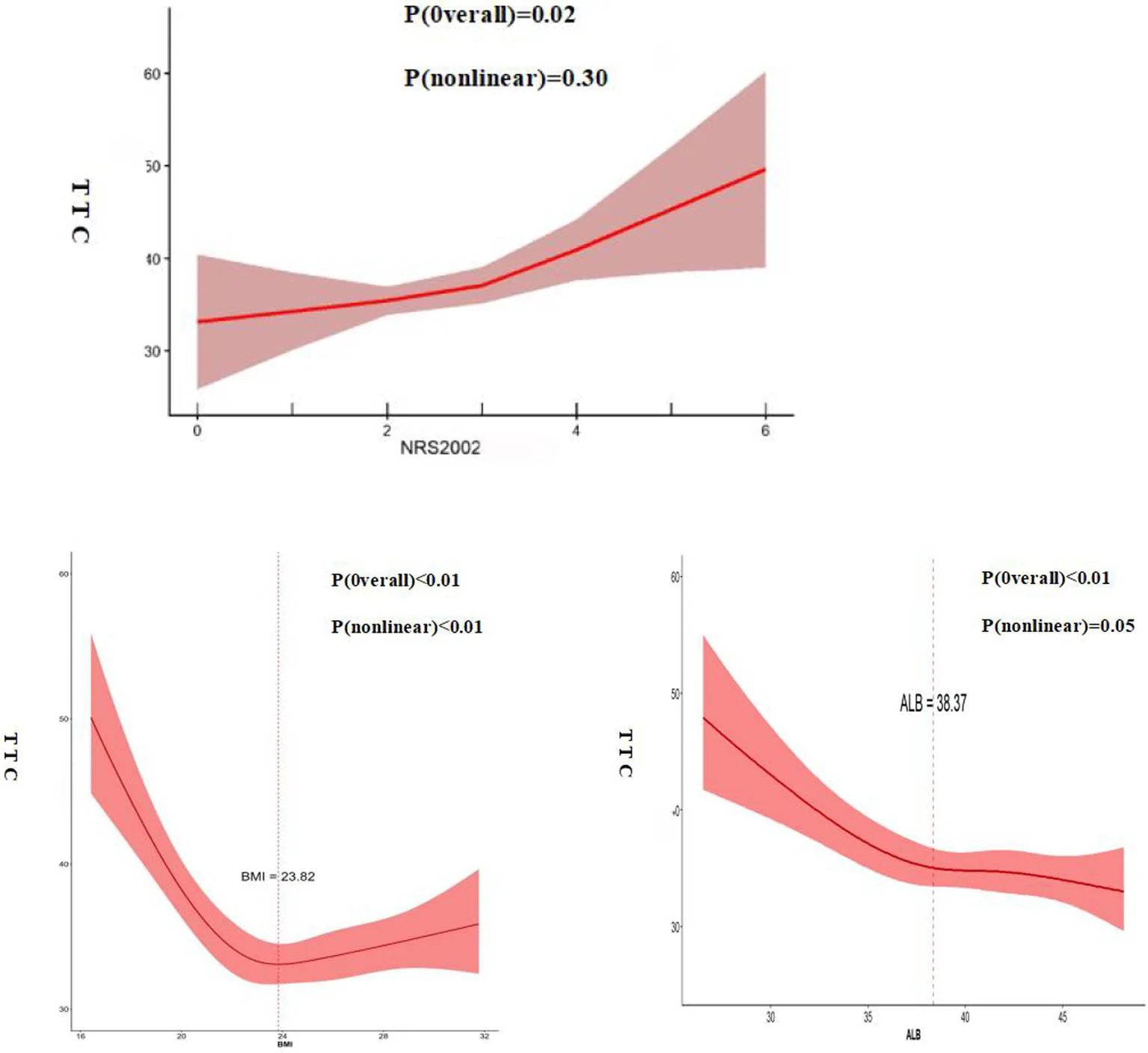
Restricted cubic spline (RCS) analyses of NRS2002, BMI and serum albumin with time‑to‑chemotherapy (TTC).

There was a U-shaped relationship between BMI and TTC, with an inflection point at 23.82 kg/m^2^. Serum albumin concentration showed an inverted L-shaped relationship with TTC, with an inflection point at 38.37 g/L, whereas the NRS-2002 score had a linear relationship (Figure 2).

### 3.3 Linear regression analysis of TTC with BMI, serum albumin, and NRS-2002 score

Multivariate linear regression analysis adjusted for age, time to postoperative resumption of diet, HGB level, operation duration, intraoperative blood loss, postoperative length of stay, and total length of hospital stay (Table 4) revealed that each 1-point increase in the NRS-2002 score was associated with an average delay of 2.36 days in the initiation of first postoperative chemotherapy (95% CI: 0.52–4.20; *p* = 0.01). BMI ≥ 23.82 kg/m^2^ and serum albumin concentration ≥ 38.37 g/L were associated with an approximately 4-day earlier initiation of chemotherapy.

**Table 4:** Linear regression analysis.

	Variable	β(95%CI)	*T*	*P*
NRS2002		2.36(0.52 ~ 4.20)	2.48	0.01^*^
BMI	<23.82(328)			
	≥23.82(372)	−4.38 (−6.64 ~ −2.12)	−3.81	<0.01^**^
ALB	<38.37(245)			
	≥38.37(455)	−4.22(−6.87 ~ −1.57)	−3.12	<0.01^**^

**P < 0.01, statistically significant difference; *p < 0.05, statistically significant difference.

### 3.4 Logistic regression analysis of TTC in relation to BMI, serum albumin concentration, and NRS-2002 score

Multivariate logistic regression analysis (Table 5) revealed that adjusting for age, time to postoperative resumption of diet, hemoglobin level, operative duration, intraoperative blood loss, postoperative stay and total hospital stay, every 1-point increase in the NRS-2002 score led to a 57% increased risk of delayed postoperative chemotherapy (≥6 weeks) (OR = 1.57, 95%CI: 1.18–2.09; *p* < 0.01). Patients with a BMI ≥ 23.82 kg/m^2^ had a 42% lower risk of delay (OR = 0.58, 95%CI: 0.40–0.83; *p* < 0.01). Serum albumin concentration was not independently associated with this parameter in this model (OR = 0.79, 95%CI: 0.52–1.18; *p* = 0.24).

**Table 5:** Logistic regression analysis.

	Variable	OR(95%CI)	*p*
NRS2002		1.57(1.18 ~ 2.09)	<0.01^**^
BMI	<23.82(328)		
	≥23.82(372)	0.58(0.40 ~ 0.83)	<0.01^**^
ALB	<38.37(245)		
	≥38.37(455)	0.79(0.52 ~ 1.18)	0.24

**p < 0.01, statistically significant difference; *p < 0.05, statistically significant difference.

### 3.5 Regression analysis by gastric and intestinal cancer subgroups

There was a significant interaction effect between the NRS-2002 score and tumor type in the linear regression model (P for interaction = 0.04). and the NRS-2002 score was a significant predictor of TTC among patients with gastric cancer (*β* = 3.90, *p* < 0.01) but not colorectal cancer (*p* = 0.47). By contrast, no significant interactions of BMI and serum albumin with tumor type were detected. (Tables 6, 7).

**Table 6:** Stratified linear regression analysis results by cancer type (gastric vs. colorectal).

	Variable	Gastric		Colorectal		Pforinteraction
		β(95%CI)	*P*	β(95%CI)	*P*	
NRS2002		3.90(1.49 ~ 8.31)	<0.01^**^	0.80(−1.41 ~ 2.99)	0.47	0.04^*^
BMI	<23.82(328)					
	≥23.82(372)	−4.86(−8.94 ~ −0.79)	0.02^*^	−4.11(−6.82 ~ −1.40)	<0.01^**^	0.82
ALB	<38.37(245)					
	≥38.37(455)	−4.25(−8.32 ~ −0.31)	0.07	−3.81(−7.14 ~ −0.49)	0.02^*^	0.91

**P < 0.01, statistically significant difference; *P < 0.05, statistically significant difference.

**Table 7:** Stratified logistic regression analysis results by cancer type (gastric vs. colorectal).

	Variable	Gastric		Colorectal		Pforinteraction
		OR(95%CI)	*P*	OR(95%CI)	*P*	
NRS2002		1.78(1.11 ~ 2.95)	0.02^*^	1.43(0.99 ~ 2.04)	0.05	0.42
BMI	<23.82(328)					
	≥23.82(372)	0.59(0.33 ~ 1.05)	0.08	0.55(0.34 ~ 0.88)	0.01^*^	0.86
ALB	<38.37(245)					
	≥38.37(455)	0.72(0.38 ~ 1.33)	0.29	0.86(0.50 ~ 1.50)	0.59	0.85

**P < 0.01, statistically significant difference; *P < 0.05, statistically significant difference.

## 4 Discussion

This study employed RCS analysis to investigate the relationship between TTC and patients’ nutritional status. We found that, compared with patients in the well-nourished group, patients in the poorly nourished group had poorer early postoperative nutritional status, which was associated with delayed initiation of chemotherapy. After adjustment for all covariates, BMI exhibited a U-shaped association, serum albumin concentration showed an inverted L-shaped threshold effect, and the NRS-2002 score was linearly and positively associated with TTC; among gastrointestinal cancer subgroups, only the NRS-2002 score showed a significant interaction.

In this study, there was a positive linear correlation between the early postoperative NRS-2002 score and the TTC. For every one-point increase in the NRS-2002 score, the time to the first postoperative chemotherapy was prolonged by an average of 2.36 days, and the risk of delay increased by 57%. The NRS-2002 score is a nutritional risk screening tool recommended by the European Society for Parenteral and Enteral Nutrition (ESPEN) and is currently the most commonly used tool in clinical practice. It consists of three dimensions: disease severity score, nutritional status impairment score, and age score, with total scores ranging from 0 to 7. A score of ≥3 indicates the presence of nutritional risk. In terms of disease severity, radical surgery for gastrointestinal cancer is a major abdominal operation. Complications such as post-operative infection, wound healing disorders, and anastomotic leakage affect the postoperative recovery period, thus forcing the postponement of chemotherapy initiation (38–40). In the nutritional status impairment dimension, nutritional reserves are quantified through three objective indicators: weight change rate, BMI, and food intake. From a pathophysiological perspective, a patient’s nutritional status is closely related to substance metabolism, the functional operation of vital organs, immune response, and the stability of cell membranes. Malnutrition reduces tolerance to chemotherapy, thereby delaying chemotherapy (41). Early nutritional intervention can effectively improve a patient’s nutritional status and physiological state, and ensure smooth treatment progress. The above findings are consistent with the conclusions of Dan et al. (42). Whole-course dynamic nutritional management helps to improve patient tolerance, enhance quality of life, and achieve better prognosis (43). In terms of age, as the function of elderly patients (≥70 years old, scored 1 point) decreases and the incidence of complications increases, the recovery period is prolonged, leading to a delay in chemotherapy (44). Notably, the European Society for Clinical Nutrition and Metabolism (ESPEN) has issued evidence-based guidelines for the nutritional care of cancer patients, emphasizing the importance of nutritional screening (45). Moreover, it provides a Grade A recommendation to major surgery patients with nutritional risk, suggesting that nutritional risk should be screened immediately after the diagnosis of cancer, and a detailed nutritional assessment should be carried out when risks are present. This study further emphasizes the importance of nutritional monitoring for inpatients. The NRS-2002 score has an independent predictive value for TTC. For patients scheduled for postoperative adjuvant chemotherapy, active nutritional risk screening and timely intervention may help reduce the risk of delayed postoperative adjuvant chemotherapy.

In this study, serum albumin concentration exhibited a nonlinear inverted L-shaped relationship with TTC. As the serum albumin concentration decreased, the interval to the initiation of postoperative chemotherapy increased; when the serum albumin concentration was ≥ 38.37 g/L, the curve flattened, indicating a saturation effect, whereby further increases in serum albumin no longer markedly affected the timing of chemotherapy. Hypoalbuminemia, as both a consequence and a marker of inflammation, impairs the body’s appropriate response to surgical or chemotherapeutic insults (46). The observed saturation of the effect of albumin on chemotherapy timing at a specific inflection point may correspond to the postoperative inflammatory response. When serum albumin concentration falls below this inflection point, inadequate plasma colloid osmotic pressure fails to maintain microcirculatory stability, precipitating tissue edema and impaired lymphatic return, thereby increasing the incidence of postoperative infection, anastomotic leakage, and delayed wound healing. These complications prolong postoperative recovery and constitute objective factors that necessitate the deferral of chemotherapy. By contrast, a serum albumin concentration ≥ 38.37 g/L suggests that capillary leakage has largely ceased. As a subjective screening instrument, the NRS-2002 may differ from objective biochemical markers; once the score is ≥3, it is difficult for the scale to precisely reflect the severity of malnutrition. Our findings identify an exploratory inflection point at a serum albumin concentration of 38.37 g/L, around which the association with the timing of initial postoperative adjuvant chemotherapy appears to change. A screening strategy of “NRS-2002 initial screen + ALB confirmation” may help more accurately identify patients at high risk for chemotherapy delay. Whereas the conventional inflection-point-related threshold for hypoalbuminemia is 35 g/L (31), this study generates a hypothesis that for patients scheduled for postoperative adjuvant chemotherapy, the value of 38.37 g/L may serve as a suggestive reference for perioperative nutritional intervention rather than a validated clinical target. Patients with early postoperative high nutritional risk and a serum albumin concentration < 38.37 g/L warrant active clinical attention to potentially reduce the risk of missing the postoperative chemotherapy window.

In this study, BMI was found to have a “U”-shaped relationship with TTC. This finding suggests that both excessively high and low BMIs can affect TTC after surgery, which is consistent with previous research indicating a “U”-shaped relationship between BMI and long-term tumor prognosis. Optimizing BMI is associated with improved long-term survival rates (47). In this study, compared with patients with low body weight, those with a BMI ≥ 23.82 kg/m^2^ had a 42% lower risk of delayed chemotherapy and initiated chemotherapy 4 days earlier. A higher BMI often indicates better nutritional status, nutritional reserves, and immune substrates, which are beneficial for buffering metabolic stress caused by surgical trauma and thus accelerating functional recovery (48). However, it is worth noting that an excessively high BMI is still associated with other life-threatening risks, such as infection and thrombosis (49), which can affect recovery and delay chemotherapy. Therefore, although an elevated BMI may confer certain short-term treatment advantages, an excessively high BMI should not be considered an ideal state. A relatively low BMI implies a risk of malnutrition. Clinically, a low BMI (especially <18.5 kg/m^2^) is often accompanied by cancer-related sarcopenia, leading to low immune function, increased infection risk, increased complications, and a prolonged hospital stay. These complications cause a chain reaction that delays postoperative recovery and forces the postponement of chemotherapy (50–52). This study further emphasizes that for patients scheduled for postoperative adjuvant chemotherapy, more attention should be given to individualized BMI management in clinical practice. Maintaining nutritional status and preventing sarcopenia are beneficial for improving prognosis (53). During the perioperative period, comprehensive attention should be given to patients. Through weight management and nutritional support, may help reduce the risk of chemotherapy-related delay.

Early postoperative nutritional status is strongly associated with the timing of adjuvant chemotherapy initiation. Poor postoperative nutrition tends to exacerbate systemic inflammation, delay gastrointestinal recovery and wound healing, and weaken patients’ physical tolerance. These unfavorable physiological changes increase the risk of chemotherapy-induced toxicity and surgical complications (54). With respect to clinical safety considerations, oncologists usually delay chemotherapy delivery for patients with suboptimal nutritional status. As a critical marker of nutritional deficit, hypoalbuminemia indicates depleted visceral protein reserves. Such metabolic disturbance may alter the *in vivo* distribution and clearance of chemotherapeutic agents, further increasing treatment-related risks (55). Moreover, malnutrition suppresses host immune function, increasing patient susceptibility to postoperative and chemotherapy-associated infections (56). Overall, impaired nutrition, excessive inflammatory activation, and compromised immune defense jointly contribute to deferring the initiation of adjuvant chemotherapy. From a practical clinical perspective, malnourished patients commonly present with fatigue, anorexia, and decreased physical activity. These clinical manifestations substantially influence multidisciplinary decisions on the appropriate timing of chemotherapy initiation. Considering the inherent limitations of this single-center retrospective study, further prospective investigations incorporating systematic inflammatory and immune biomarkers are needed to validate the underlying biological mechanisms.

We found significant differences between gastric cancer and colorectal cancer in terms of the effect of nutritional status on the timing of postoperative chemotherapy. The NRS-2002 score for TTC was tumor type specific and had an independent predictive effect on gastric cancer but not on colorectal cancer. This difference may be related to anatomical and physiological characteristics and may be associated with the structure and digestive and absorptive functions of the stomach. Patients with gastric cancer often experience a decrease in appetite and a weakening of digestive and absorptive functions in the early stage, accompanied by varying degrees of digestive dysfunction, making them more prone to malnutrition. According to statistics, the incidence of malnutrition in gastric cancer patients is as high as 87%, ranking among the top digestive system malignancies (57). By contrast, the intestine has a strong compensatory ability. Although patients with colorectal cancer have different disease locations and clinical symptoms, the main manifestations are abdominal masses, hematochezia, and changes in stool properties and shape. There are fewer cases of decreased appetite and weight loss, and the impact on overall nutritional status is delayed in the early stage (58). Therefore, the sensitivity of the NRS-2002 score is reduced. By contrast, the protective effect of BMI on TTC was consistent in both groups, suggesting that BMI, as an indicator of nutritional reserve, is universally applicable across different cancer types and is not affected by tumor location. The predictive effect of serum albumin was significant only in patients with colorectal cancer and marginally significant only in patients with gastric cancer. This phenomenon may be related to the crucial role of albumin in maintaining intestinal barrier function, preventing bacterial translocation, and reducing postoperative infections; however, this mechanism is relatively less important in gastric cancer (59). The above findings suggest that differential nutritional assessment strategies should be developed in clinical practice: for patients with gastric cancer, comprehensive nutritional screening (NRS-2002) in the early postoperative stage should be strengthened, with a focus on food intake, weight changes, and digestive function; for patients with colorectal cancer, in addition to monitoring BMI, serum albumin levels should be closely tracked, and BMI should be maintained within an appropriate range.

## 5 Limitations of the study

This study has several inherent limitations. Current clinical guidelines specify consistent time frames for initiating postoperative adjuvant chemotherapy irrespective of tumor stage. Although pathological stage was not included in our statistical models, surgical duration and intraoperative blood loss were used as surrogate markers to reflect surgical complexity and postoperative recovery difficulty, ensuring the basic reliability of our analytical results. Even so, these intraoperative metrics cannot fully substitute for comprehensive clinical evaluation. Key detailed confounders, including precise TNM grading, stoma formation status, postoperative complication severity grading, and detailed pathological features, neoadjuvant therapy history, baseline performance status, individualized chemotherapy regimens and patient treatment preferences, were not fully incorporated into the analysis. These unmeasured clinical variables are critical determinants of TTC, and the use of intraoperative surrogate indicators cannot completely offset the absence of these data, inevitably resulting in residual confounding bias. Our results should be interpreted as exploratory observational associations rather than causal conclusions. A further notable limitation originates from our enrollment strategy, which exclusively included patients who ultimately received adjuvant chemotherapy and excluded individuals with severe postoperative complications. Since severe surgical complications constitute a major clinical cause of delayed chemotherapy initiation, this exclusion criterion may introduce prominent selection bias. Patients with severe complications usually suffer from impaired nutritional recovery and markedly prolonged TTC. Therefore, our findings are applicable only to patients with relatively uneventful postoperative courses, and the generalizability to high-risk surgical populations remains limited. Moreover, several potential influencing factors, including inflammatory biomarkers (CRP and IL-6), were not analyzed in this study. Their potential confounding effects on TTC remain to be further investigated. As a single-center retrospective study, this work is prone to inherent selection bias and a constrained sample size. The limited sample size for subgroup analyses may decrease the statistical power; hence, comparative results between gastric and colorectal cancer cohorts should be interpreted with caution. Inflection points identified (BMI 23.82 kg/m^2^, albumin 38.37 g/L) are exploratory findings specific to our dataset and have not been externally validated. Therefore, they should be interpreted with caution and not directly applied as clinical cut-offs in routine practice. Future large-scale, multicenter prospective studies are needed to validate our conclusions. Combining genomic and immune biomarkers with rigorous analytical strategies, such as competing risk models, causal inference approaches, and externally validated machine learning algorithms, can help develop standardized, evidence-based clinical criteria to optimize postoperative adjuvant chemotherapy timing.

## 6 Conclusion

This study revealed that postoperative nutritional status is associated with TTC. On the basis of the restricted cubic spline model, a nonlinear association between early postoperative nutritional status and the initiation time of adjuvant chemotherapy for gastrointestinal cancer was revealed. Specifically, BMI and serum albumin had U-shaped and inverted L-shaped relationships with TTC, respectively, whereas the NRS-2002 nutritional score was linearly and positively associated with TTC. In clinical practice, implementing enhanced recovery after surgery and strengthening nutritional risk screening are potential strategies that warrant investigation of the delay of the first round of chemotherapy. These measures help to identify patients at risk of chemotherapy delay early, actively intervene, integrate them into a multidisciplinary collaboration, and construct a more comprehensive prediction-intervention system to achieve precise and individualized perioperative management for cancer patients. Given the retrospective single-center design, the inflection-point values of BMI and serum albumin derived from this study are hypothesis-generating findings and should not be regarded as validated clinical targets for routine clinical application. Further studies are required to validate these findings.

## Data Availability

The original contributions presented in the study are included in the article/supplementary material, further inquiries can be directed to the corresponding author.
